# Review of the Current State of Optical Characterization and Design of Electronic States in Plasmonic Materials—From Noble Metals to Silverene and Goldene

**DOI:** 10.3390/nano15201548

**Published:** 2025-10-10

**Authors:** Rosen Todorov, Temenuga Hristova-Vasileva

**Affiliations:** 1 Institute of Optical Materials and Technologies “Acad. J. Malinowski”, Bulgarian Academy of Sciences, Acad. G. Bonchev Str., bl. 109, 1113 Sofia, Bulgaria; teddie@iomt.bas.bg; 2 Institute of Solid State Physics, Bulgarian Academy of Sciences, 72 Tsarigradsko Chaussee Blvd., 1784 Sofia, Bulgaria

**Keywords:** plasmonics, intermetallics, localized surface plasmon resonance, charge transfer, hot electrons, optical properties, SERS, photocatalysis, 2D material plasmonics, UV plasmonics

## Abstract

Materials’ plasmon activity is defined by their electronic structure. Nowadays, the application of plasmonic materials is increasingly determined by the possibilities to control the electronic processes in them. The electronic structure’s design is of particular importance for tuning the plasmon frequency and the excitation of hot electrons, which are important parameters determining the interaction of the nanostructures with the environment. The effective control of these parameters is important for the improvement of the efficiency and sensitivity of various processes, diagnostic methods and technologies in the field of photocatalysis and surface enhancement spectroscopies. This review is focused on the characterization techniques and the approaches for tuning the electronic states of plasmonic media. The diversity of materials and their electronic structure determine the approach for the engineering of the electronic structure. In the case of noble metals, the possibility for tuning the energy for interband transitions from their *d* band is considered by using intermetallic alloys (between noble metals themselves and with an addition of post-transition metals in them), while in semiconductor materials—the effect of charge transfer is mainly used. Such knowledge is not only essential from a practical point of view, but also contributes to understanding the processes in the field of new materials such as 2D noble metals and intermetallics.

## 1. Introduction

**Figure 1:**
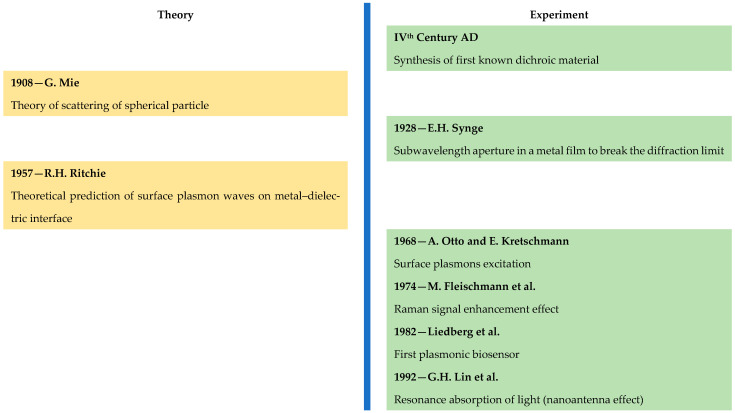
Schematic presentation of theoretical and experimental findings until the year 2000, which form the basis of contemporary plasmonics [13,14,15,16,17,18,19,20,21,22].

Nowadays, plasmonic materials find application in various devices, used in sensorics [1,2,3], biodiagnostics [4,5,6], energy harvesting [7,8,9,10] and non-linear optics [1,2,3,4,5,6,7,8,9,10,11,12]. All applications are based on interaction of electromagnetic waves from the optical range with electrons from the material on a continuous metal–dielectric surface or on the surface of a nanoparticle, causing the well-known phenomena of surface plasmon resonance (SPR) and localized surface plasmon resonance (LSPR). Behind all these there is a number of theoretical and experimental findings, as well as a process of developing new plasmonic materials, which started after the new millennium began, and which also will be considered in the present review (Figure 1).

The beginning of the scientific research in the field of plasmonics was set to be the year 1957, when R.H. Ritchie [16] predicted the distribution of electronic waves (surface plasmons) at a smooth, semi-infinite metal–vacuum boundary. The first reports for experimental observation of SPR in silver films and silver diffraction gratings were published in 1968 [17,18]. Initially the plasmonic media were not exotic—in practice, they were nothing but continuous metal/dielectric interfaces of noble metals (silver and gold) deposited as thin films or patterned into gratings exposed on air. The large negative values of the real part and the small dielectric losses, related to the imaginary part, ε″, of the complex permittivity in silver and gold, determining their high efficiency as plasmonic materials in the visible spectral region, made them the first objects for investigation of “surface plasmons”. That is why, nowadays, almost every textbook on plasmonics describes mainly the application of these two metals in the scientific area [23,24].

From a historical point of view, LSPR found a practical application much earlier, in antiquity [13] and in the Middle Ages [25].

The theoretical foundations of LSPR were laid by G. Mie at the beginning of the 20th century [13]. Although he did not explicitly use the term “plasmon”, the sharp resonance peaks of the extinction cross-section curves for a spherical metal nanoparticle correspond to what we now recognize as the LSPR of metal nanoparticles. In the late 1960s and into the 1970s, the need for deposition of a thin metal layer led to an increased interest in studying the thickness dependence of the optical properties of thin metallic films [26,27,28,29,30,31]. Kreibig and Zacharias [26] applied the Mie theory to analyze and explain the appearance of an absorption maximum in the visible part of the electron energy loss spectrum of silver and gold particles. Subsequently, a number of works appeared that used spectroscopic reports of observing surface plasmons on metal particles, or the well-known LSPR, of discontinuous Cu, Ag, and Au films [26,27,28,29,30,31,32,33].

A milestone for plasmonics occurred in 1982, when Liedberg et al. [20,21] demonstrated the use of SPR for biosensing applications. Most applications of SPR in sensing technologies are based on the high sensitivity towards changes in the refractive index of the surrounding medium which can reach up to 10^−8^ RIU [34,35,36].

Most of the SPR sensors, mainly based on the widely used Kretschmann configuration, have been significantly evaluated through the years and are integrated in optical waveguides and fibre optic SPR systems by the deposition of a metallic layer along the length of optical waveguides or fibres [37,38,39,40]. Due to the flexibility in the selection of a LSPR substrate, which allows tuning of the wavelength throughout the visible spectrum, they find application in various fields of modern diagnostics and technology, as bio-molecular sensing, environmental surveillance and monitoring, food safety, clinical diagnostics and biomedicine [32,40,41].

A key aspect for the emerged interest in LSPR was not only the development of sensor techniques, but also the effect of amplification of the Raman signal of pyridine adsorbed on a roughened silver electrode [19], first observed in 1974, which marked the beginning of Surface-Enhanced Raman Spectroscopy (SERS), and subsequently of a whole family of surface enhancement techniques. This result opened a new area of application and searching for plasmonic materials. Only five years later, the first report for recording the Raman vibrations of molecules from a single cyanide monolayer on a metal surface was published [42].

In the early 1980s, Moskovits [43] summarized the main phenomena that contribute to signal amplification. These are the electromagnetic interactions between the electron gas of the plasmonic nanostructure and the analyte and the implementation of a chemical (catalytic) interaction, which on its part leads to a significant change in the polarizability of the analyte’s molecule and thus, to a much greater amplification of the Raman signal compared to the electromagnetic interaction. The use of these effects, generally called surface enhanced Raman scattering, causes the ability to register and analyze single molecules under special conditions, and makes them indispensable, especially in the field of molecular diagnostics, for the analysis of biological markers in the genome and proteome during the identification of diseases at a molecular level [44,45,46,47,48]. It forms the basis of modern techniques for molecular diagnostics such as Specific High-Sensitivity Enzymatic Reporter UnLOCKing (SHERLOCK) and DNA Endonuclease-Targeted CRISPR Trans Reporter (DETECTR), and has its prospect in gene-editing tool SERS-based CRISPR/Cas (SERS-CRISPR) [46]. The Raman signal enhancement effect not only enables the development of new sensing and diagnostic techniques, but is also the foundation of a totally new field of enhanced spectroscopies, which nowadays include Surface-Enhanced Raman Spectroscopy (SERS), Surface-Enhanced Fluorescence (SEF), Surface-Enhanced InfraRed Absorption (SEIRA), Surface-Enhanced Hyper Raman Spectroscopy (SEHRS) and Surface-Enhanced Raman Optical Activity (SEROA) [49,50,51,52].

Since the fluctuations in the electronic plasma on the surface of the metallic structures determine the enhancement of the electromagnetic field and Mie’s theory lacks sufficiency to describe the plasmonic behaviour of an ensemble of particles, the question about the interaction between two or more particles appears [53,54,55,56]. These investigations lead to the development of a nanoantenna for optoelectronics, which was first used by G.H. Lin et al. for the realization of a resonance absorption of light [22]. With the increasing usage of electron beam lithography in the early 2000s, the scientists focused their research on the design of metallic nanostructures (like bowtie antennas, nanorods, and nanoparticle dimers), which act as optical antennas that concentrate light into tiny volumes by creating “hot spots”. The idea of nanoantenna interaction is increasingly spread in various techniques and contributes to the development of surface-enhanced spectroscopies [57,58] and near-field imaging [59,60].

In the early 2010s, the plasmonic materials were still limited to the metals family: Au, Ag, Cu, Pd, Pt, Al, some noble metal alloys (Au-Ag, Au-Cu, Ag-Cu) and highly doped semiconductors (Si, indium tin oxide (ITO), CuS) [61]. In order to fulfil the needs of the modern techniques in the fields of solar cells and biodiagnostics, the necessity for the development of materials which possess plasmonic properties not only in the visible spectrum, but also in a wider part of the infrared (graphene and graphene oxides, transition metal chalcogenides, nitrides and MXenes [62,63,64,65,66,67,68]) and the ultraviolet (post-transition (*p*-block) metals and their alloys [69,70,71,72,73,74]) spectral regions increases.

The electronic structure of these plasmonic materials not only determines the spectral range of application, but also governs their interaction with the environment. This interaction determines the processes of excitation of hot electrons, through intraband or interband transitions, in the plasmonic materials and the charge transfer processes between the individual nanostructures and/or the analyte [75,76]. In the present, even though this is in a very early stage, there is an increasing number of studies focused on charge transfer to the HOMO or LUMO levels of organic compounds and, respectively, to the conduction or valence band of inorganic semiconductors. These electronic processes are of particular significance for the application of plasmonic materials in photocatalysis and hydrogen storage [76,77], for selectivity control in the case of plasmonic sensors [77,78] and surface-enhanced spectroscopic techniques [79], which will better the sensitivity and selectivity of molecular diagnostics’ methods.

The present review paper focuses on the state-of-the-art of electronic properties’ design in plasmonic materials and the techniques for their investigation. The strategies for engineering of the electronic structure and electron transfer in different groups of plasmonic materials are also reviewed and evaluated as promising approaches for creating new materials for plasmonics.

## 2. Electronic Structure, Hot Electrons, and Nanoantenna Effect

In this section, we will briefly consider the dependencies that determine the dielectric response of materials when they interact with an external electromagnetic field, and the effects that underlie the design of the electronic structure of plasmonic materials.

### 2.1. Band Structure and Complex Permittivity

The solution to Maxwell’s equations for a wave propagating on a surface determines the conditions under which surface electron waves can propagate along a metal–dielectric interface. A detailed description of different cases—a single metal–dielectric interface, a thin metallic layer sandwiched between two dielectric media, and a spherical metal nanoparticle in a dielectric medium can be found in [80]. In the case of a continuous metal–dielectric interface, the obtained solutions for the existence of an electromagnetic wave show that the relative permittivity, *ε_r_*, must be negative and to satisfy the condition *ε_r_* > −1 for this surface, while the imaginary part *ε*″ determines the attenuation of this wave [80]. The frequency of the surface plasmon resonance (SPR), *ω_sp_* is determined by

(1) $$\omega_{sp}=\frac{\omega_{p}}{\sqrt{\varepsilon_{r}+1}}\;,$$

where ω*_p_* is the bulk plasmon frequency.

When solving Laplace’s equation in the case of a spherical metal particle with radius R and relative permittivity *ε_r_*, set in a medium with a dielectric permittivity ε_0_, the resonance conditions are given by the equation *ε_r_* = −*ε*_0_ (*ℓ* + 1)/*ℓ*, where *ℓ* is an integer and is related to oscillations of multipole moments of the electronic gas in the metal nanoparticle (*ℓ* = 1 accounts for a dipole mode; *ℓ* = 2 for quadrupole oscillations, etc.). The frequency of the localized surface plasmon resonance (LSPR), *ω_ℓ_* is

(2) $$\omega_{l}=\omega_{p}{\left[\frac{l}{\varepsilon_{0}\left(l+1\right)+l}\right]}^{\frac{1}{2}},\;\ell \;=\;1,\;2,\;3\ldots .$$

In both cases considered above—of SPR and LSPR, the complex permittivity determines their frequency, and is defined by the electronic structure of the materials. In the case of noble metals—Cu, Ag, Au, it is caused by the overlapping of the valence and conduction bands, as the valence band of these materials consists of the last *s* electronic state, located at the Fermi level and the *d* band, situated below it. The complex permittivity is determined by the electron transitions at these electron levels—intraband transitions of *s* electrons to empty *s*-*p* states above the Fermi level and interband transitions of electrons from the *d*-level to the Fermi level. Therefore, one of the most commonly used models to describe the dispersion of complex permittivity is the well-known Drude–Lorentz model. It consists of two parts: the Drude dispersion formula, *ε_D_* which takes into account the contribution of the *s* electron transitions, and a Lorentz component, *ε_L_* to take into account the contribution of the interband transitions from the *d* level to the Fermi level [81]:

(3) $$\varepsilon =\varepsilon_{D}+\varepsilon_{L}=\varepsilon_{\infty }-\frac{{\omega_{p}}^{2}}{{\omega_{p}}^{2}+i\omega \Gamma_{p}}+\sum \frac{f_{j}\omega_{Lj}^{2}}{\omega_{Lj}^{2}+\omega^{2}-i\omega \Gamma_{Lj}}\;,$$

where *ε_∞_* is the permittivity at infinite frequency; *ω_p_* is the bulk plasmon frequency and Γ*_p_* is a damping coefficient. The Lorentz-like parts are characterized by resonance frequencies *ω_Lj_*, oscillator strengths *f_j_* and damping factors Γ*_L__j_*. The plasmon frequency depends on the free electron density, *N*, the electron charge, *e* and the effective mass, m*:

(4) $$\omega_{p}^{2}=\frac{N_{c}e^{2}}{m*\varepsilon_{0}}\;,$$

where ε_0_ = 8.854 × 10^−12^ F·m^−1^ is the vacuum permittivity.

**Figure 2:**
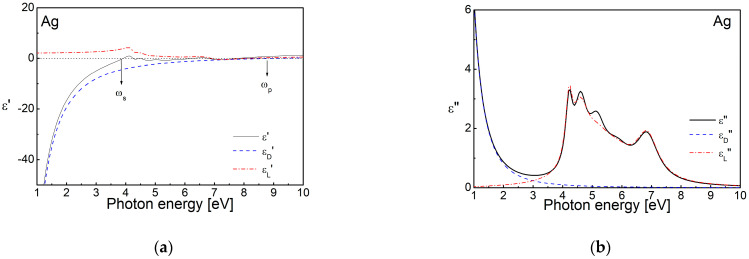
Dispersion of the real (**a**) and imaginary (**b**) parts of the complex permittivity of silver and their corresponding Drude and Lorentz components calculated by Equation (3) and the dispersion parameters, published in Ref. [82].

Figure 2 presents the real and imaginary parts of the dielectric function ε of a thin silver film, as well as the Drude and Lorentz components, calculated from the dispersion parameters, published in Ref. [82]. It can be seen that the contribution of the free electrons (i.e., the Drude component, *ε_D_*′) to the real part, *ε*′, is always negative and changes its sign at the plasma frequency, ω_p_. The positive values of the Lorentz component, *ε_L_*′, lead to an increase in *ε*′ towards the positive values, and, respectively, towards a change in the sign at a frequency smaller than *ω_p_* or the so-called screen plasmon frequency, *ω_s_*_._ At photon energies lower than this needed for interband transitions from the *d* level, the Drude component, i.e., the contribution of the free electrons, is dominant. The Lorentz component depends weakly on the photon energy at these frequencies and can be considered as a constant.

**Figure 3:**
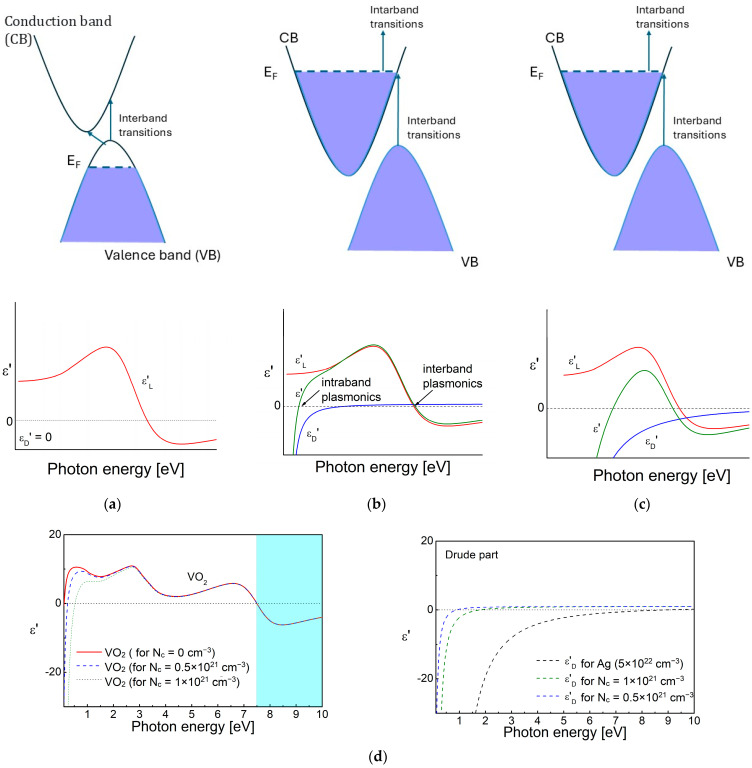
Schematic presentation of the band structure and the expected path of the complex permittivity, as well as its Drude and Lorentz components (shown as one oscillator): (**a**) pure dielectric; (**b**) conducting oxide; and (**c**) semimetal; (**d**) sketch showing the plasma tuning approach in case of a larger contribution of interband transitions, valid for all conducting oxides and semiconductors: real part of the permittivity of VO_2_ (data from Ref. [83] were used for free electron densities), where the filled area in the spectral range 7.5–10 eV shows the region of interband plasmonics because only intensive interband transitions occur, as well as different Drude curves, corresponding to different concentrations of free charge carriers N_c_ (average N_c_ values from [84] were used, where it was shown that the N_c_ in VO_2_ increases from 10^19^ to 10^23^ cm^−3^ during heating from 20 to 90 °C), showing the insignificant contribution of free electrons in the spectral range 1–10 eV (the Drude component of the complex permittivity of silver is given for comparison).

The influence of the materials’ band structure on the complex permittivity is shown in Figure 3a–c. The scheme is valid in the cases of dielectrics, conducting oxides and nitrides with various free electrons’ concentrations, and semimetals. The number of free charge carriers is smaller in semiconductors, which is a reason for the shifting of the plasma frequency towards the lower photon energies, which fall into the infrared (IR) and THz spectral regions. As a consequence, the *ε_D_*′ curve is also shifted towards the smaller frequencies. A representative case of VO_2_ [83,84], valid for all semiconductors and conducting oxides, is shown in Figure 3d. It can be seen that at photon energies higher than the resonance frequency for interband transition, the complex permittivity changes its sign and becomes negative—this is the so-called interband plasmonics. A similar effect is observed in the case of semimetals, Bi and Sb, for the near-infrared (NIR) and visible spectral regions [85]. The relatively higher free electron density, *N*_c_ up to ~10^19^–10^22^ cm^−3^, increases the contribution of the Drude component in the conducting oxides (ITO, CdO, Al:ZnO (AZO), Ga:ZnO (GZO)) [66,68,86,87] and nitrides (TiN, HfN) [65] and they possess negative values in the IR region in the range 0.5–1.5 eV.

By having information about the complex permittivity, one can model plasmonic nanostructures using various theoretical models and mathematical methods, and then proceed to their synthesis. Mie’s theory, previously already mentioned, is widely applied today for the modelling of metallic nanoparticles, including those with shells from another material—other metals, oxides and transitional metals’ chalcogenides [88,89,90,91,92,93,94,95,96,97,98]. With the development of the computational technologies, theoretical modelling is increasingly being used through Density Functional Theory (DFT) calculations or first principles (ab initio) calculations, which give information for the electronic density of states and the possibility for the prediction of the complex permittivity’s theoretical spectrum [96,97,98,99,100,101]. The main problem in the conventional DFT calculations is the correct description of the highly localized, strongly correlated *d* or *f* electrons of the transition metal and rare-earth elements. The DFT+U method is used in such cases when a Hubbard on-site Coulomb correction [102,103,104,105] is applied. It ensures a better determination of the band gaps and the magnetic moments for transition and rare-earth metals and their compounds.

The use of the Finite-Difference Time-Domain (FDTD) method, the discrete dipole approximation method and the DFT allows for the calculation of the plasmonic response, the charge distribution and the intensity of the electric field inside and around metallic structures [106,107].

### 2.2. Hot Electrons and Charge Transfer

The electronic transitions play an important role in the plasma activity of noble metals and the way they interact with the environment. These processes are the subject of research in a number of works related to the use of plasmonic structures in photocatalysis or SERS [108,109,110,111,112].

**Figure 4:**
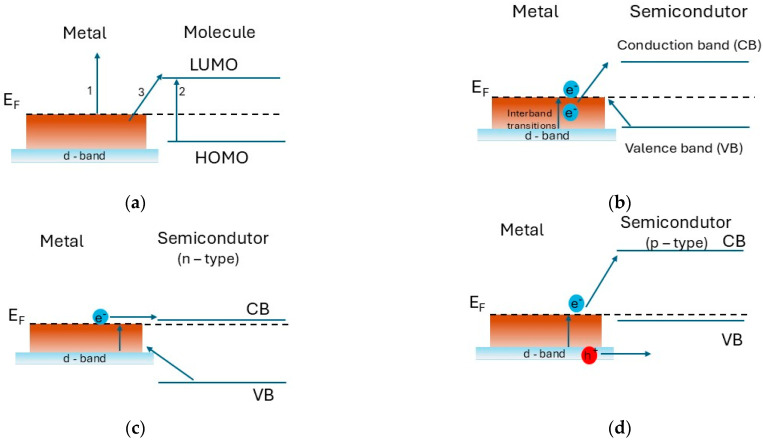
Electron transitions related to a charge transfer between (**a**) metal–organic molecule (electron transitions in SERS are marked with numbers: 1. chemical enhancement in basic state, 2. resonant Raman enhancement, 3 charge-transfer resonant Raman enhancement); (**b**) metal–semiconductor—the appearance of a Schottky barrier and charge transfer in the case of an undoped semiconductor are shown; (**c**) n-type semiconductor; and (**d**) p-type semiconductor. The figures are adapted and generalized from those presented in Refs. [76,78,108].

The possible electron transition processes between an organic molecule and plasmonic nanostructures made of metal or semiconductor are considered in Ref. [78]. In the case of noble metals, the intraband transitions involve the excitation of an *s* electron to the vacant *s-p* levels above the Fermi level (Figure 4a,b). The hot electrons obtained from these transitions are non-thermally distributed above the Fermi level, leaving holes below it. They can move to an antibonding orbital (or to a lower unoccupied molecule orbital (LUMO) in the case of interaction with an organic molecule) and lead to decomposition of the molecule [76].

The interband transitions cause excitation of electrons in the *sp* band near the Fermi level and the formation of high-energy holes in the *d* band well below the Fermi level, accordingly. Their energy is determined by the energy exceeding the threshold, required for such interband transitions, and the photon energy. In the case of a metal–semiconductor transition, a Schottky barrier appears, in which the charge transfer depends on the width of the bandgap and the conductivity type of the semiconductor (Figure 4b–d).

In addition, the type of laser radiation matters in charge transfer, especially in the case of picosecond lasers, whose pulse duration coincides with the electron–phonon interactions, while for the femtosecond lasers it coincides with the electron–electron interactions [76]. Therefore, by controlling the electron transitions we can determine the charge transitions of the bonding or antibonding molecular orbitals, which determine the chemical reactions and selectivity of the interaction of the plasmonic structure. This can find application in the field of surface-enhanced spectroscopy techniques (e.g., SERS), biodiagnostics [108], catalysis, and hydrogen storage [76].

### 2.3. Nanoantenna Effect

The research on metallic nanoparticles has raised the question of how the plasmon waves propagating on their surface interact with each other. This led to the idea of the nanoantenna effect, which initially found its application in electronics. The idea arose in the process of research aimed at improving the efficiency of solar cells. It started in the early 1970s, when Robert L. Bailey developed and patented a converter of electromagnetic radiation from the infrared and visible spectral regions directly into electrical energy [113]. His transducers consisted of triangular or conical metal elements of a few centimetres in size. Later, in 1984, Alvin M. Marks patented sub-micron structures that serve to convert light [114]. The concept of the role of LSPR of a single particle led to the question of how the plasmon waves of two neighbouring or of an ensemble of particles interact [53]. The idea of controlling light propagation through nanoantenna regions was given by Guang H. Lin et al. [22,115], who published in 1996 their results on the observation of a resonant absorption of light with photon energy smaller than the band gap of the materials, by which the investigated nanostructures were prepared. In addition, they also presented a fabricated parallel dipole nanoantenna array for the first time.

A nanoantenna effect between two nanoparticles of noble metals can be realized when they are close enough to each other and support LSPR after irradiation with light. Then, due to the interaction of the electrons’ oscillations on their surface, new hybrid modes are created. An extreme amplification of the electric field is observed in the space between the particles or, as is generally accepted, it becomes a hot spot.

The nanoantenna effect is the reason new phenomena are to be observed:

- Subwavelength light localization—the light is focused on volumes much smaller than the wavelength, as by this way the diffraction limitations are suppressed, and a nanoscale optical control is achieved [116];

- Directional emission and scattering—the interacting metallic particles can act like a radio antenna, emitting or scattering electromagnetic radiation in a specific direction falling in the optical frequency range [117];

- Quantum and tunnelling effects—due to the minimal distances between the nanoparticles, of the order of several tens of nanometers, charge carriers could jump between them through quantum tunnelling, and alter the plasmonic behaviour by producing charge transfer plasmons. This property of nanoantennas is particularly important in ultrafast optical switching and nano-optoelectronic devices [118].

The possibility for tuning the nanoantennas’ resonance frequency across the visible and near-infrared spectrum by adjusting the particle size, shape, and gap distance is an important advantage. The studies show [117] that the shape of the antenna is an important parameter, as particles in the shape of triangles and nanorods concentrate light much more effectively than a sphere.

The nanoantenna effect finds application in plasmonics in two main directions: (1) it is used in various ultra-high-resolution methods, such as near-field optical microscopy, micro-Raman, micro-infrared spectroscopy [117,119,120,121], and (2) the control of light propagation and the polarization state underpin the polarimetric methods for the characterization of plasmonic nanostructures [122].

The nanoantenna effect is fundamental for photovoltaics [123], quantum optics and gen communication technologies [124,125]. The optical tweezers technique [126], in which the nanoantenna effect is used for optical trapping during the investigation of biological objects, uses the effect of a highly focused laser beam to apply and measure pN (piconewton)-sized forces on micron-sized dielectric objects, such as small clusters or living cells. This method allows a precise manipulation and measurement of these objects under a microscope. Developed by Arthur Ashkin in the 1970s, the optical trapping has found applications in various fields, including physics and biology, from atomic cooling to DNA unzipping [127].

## 3. Techniques for Characterization of Plasmonic Materials and Nanostructures

**Figure 5:**
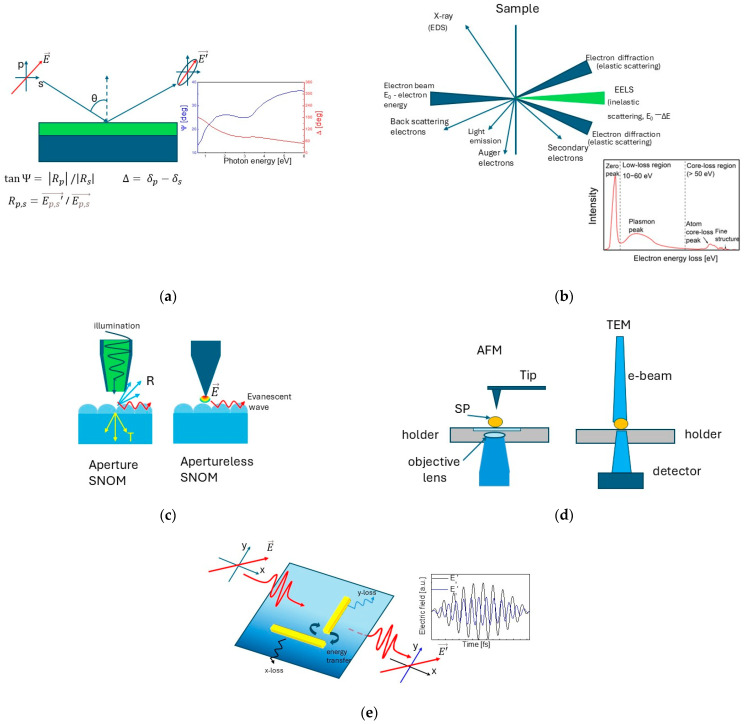
Roadmap of the experimental techniques for the investigation of electronic processes in plasmonic nanostructures: (**a**) Spectroscopic Ellipsometry; (**b**) Electron Energy Loss Spectroscopy (EELS); (**c**) Scattering Near-field Optical Microscopy (SNOM); (**d**) Single Particle (SP) Spectroscopy, (**e**) Interferometric and Polarimetric Microscopy.

Below we will focus on the techniques for the characterization of the optical properties of plasmonic structures and providing information about the electronic processes in them (see Figure 5). These techniques include those which provide information about complex permittivity, such as spectral ellipsometry and Electron Energy Loss Spectroscopy (EELS), and methods that directly visualize the behaviour of electronic processes (different plasmonic modes, charge transfer, etc.) in nanostructures, such as scattering near-field optical microscopy and Single Particle Spectroscopy. It should be noted that the mentioned techniques can work in combination with each other. General information about the applicability of these methods, as well as the possibilities of their combination, and the properties and constants that can be evaluated, are presented in Table 1.

### 3.1. Spectroscopic Ellipsometry

Spectroscopic Ellipsometry is one of the first methods used for the investigation of the optical properties of plasmonic materials. The method’s principles are based on the analysis of the change in the polarization state of light upon interaction with the surface of an object under study. The grounds of ellipsometry were laid by P. Drude [128,129], who not only theoretically developed the ellipsometry’s basic equations, but also experimentally investigated the reflection of light from the surface of solids for the first time and performed the first ellipsometric experiments for the determination of optical constants of metals [129].

Ellipsometry, which mainly uses reflectance measurements to determine the dielectric response of highly absorbing coatings, is very suitable for plasmonics. This is due to the fact that the metals, which are the main materials in plasmonic nanostructures, have high absorption coefficients over almost the entire optical range, which makes them opaque and therefore using transmittance measurements is impractical except for very thin layers [130]. Furthermore, the high surface roughness, which scatters the light strongly, as well as the luminescence from the investigated sample can significantly influence the measured ellipsometric quantities and their analysis, accordingly.

Nowadays, various ellipsometry instruments allow the investigation of nanostructures in a wide spectral interval—from the ultraviolet at a photon energy of 97 eV (λ = 12 nm) [131] to the far infrared and part of the THz spectral ranges, up to 0.5 THz (λ = 500,000 nm) [132]. The main difficulty of this is the preparation of polarizing elements that have the necessary properties to control the polarization of light in a very wide spectral range, and therefore different ellipsometric systems are needed to cover the above-mentioned spectral range [133].

The first ellipsometric measurements of thin films with a discontinuous structure date to the early 1970s in the works of T. Yoshida and T. Yamaguchi [28]. The capabilities of the existing instruments at that time allowed only the use of single-wavelength measurements. The appearance of the Spectroscopic Ellipsometry as we know it today, in the second half of the 1970s [128,130], set a new stage in plasmonic materials’ investigations.

A number of studies can be found in the scientific publications, related to the ellipsometric characterization of various plasmonic materials—metal nanostructures, noble metals and their alloys [134,135,136,137,138], nitrides [139] and 2D materials (graphene, MXene) [140,141,142]. This wide interest is determined by some general advantages that Spectroscopic Ellipsometry has. It offers the capability for the determination of phase change differences with an accuracy comparable to that of interferometric techniques, which makes this method particularly sensitive in studying processes related to changes in the thickness of thin films, periodic nanostructures and surface phenomena [143].

The sensitivity of Spectroscopic Ellipsometry and the good accuracy in determining the complex permittivity of thin-film coatings is the reason why it is widely used to study the influence of the thickness of thin layers of noble metals (Ag and Au), which are used as plasmonic materials or participate in the construction of plasmonic structures with thicknesses between 5 and 50 nm [144,145,146]. The usage of the effective media approximation theory, EMA (detailed information about the different theoretical models for the description of the properties of an effective medium made up of two or more different phases can be found in [147]), in the analysis of ellipsometric measurements, allows the determination of the threshold thickness at which the thin film coatings transition from a continuous form to a discontinuous medium made up of individual metal aggregates. In addition, the analysis of ellipsometric measurements of very thin films can demonstrate a change in the values of the imaginary part of ε depending on the size of the metal grains [144,145], due to the so-called size effect, in which the free electron path becomes smaller than the size of the metal aggregates and a corresponding change in the damping parameter is obtained. It is assumed in Equation (3) that the Γ_p_ is constant at a given temperature. When the dimensions of the nanostructures become smaller than the free electron path, the quantum size effects and the processes of charge transfer between the particles and the surrounding material start to influence the electronic relaxation. As a result, an increase in the values of the complex permittivity’s imaginary part is observed [148,149,150], while the influence of the size effect on the real part of ε is weak and becomes noticeable for nanostructures with dimensions of ~1–5 nm [149]. The information about the complex permittivity allows the determination of the transverse plasmon modes on the surface of the nanostructures, expressed by the appearance of a maximum in the imaginary part of ε, as well as the longitudinal oscillations, by using the optical loss function Im−1ε=ε″(ε′2+ε″2) [145].

Since the ellipsometric angles provide direct information about the change in the polarization and the phase of light, Spectroscopic Ellipsometry is nowadays an important technique for the analysis of anisotropic materials and metasurfaces [151,152,153]. The possibility to determine thickness changes in the picometer range, together with the ability to monitor these changes in real time, makes the ellipsometry an indispensable technique for the in situ control of thin film deposition processes and the synthesis of nanostructures [125,154,155,156]. High-resolution image ellipsometry is increasingly becoming the focus of research, because this technique allows the mapping of the studied object’s surface depending on the ellipsometric parameters, with a spatial resolution of less than 1 μm [157,158].

A key point in the case of solving the ellipsometric problem for determining the optical parameters of a system under study by using data from spectral ellipsometric measurements is the choice of an appropriate dispersion model. The most important in this choice is the electronic structure of the studied material. The Drude–Sommerfeld model, where the contribution of interband transitions is constant, was used in earlier works [31]. This model works well in the region where the contribution of free electrons dominates and starts to give deviations from the real values at intensive interband transitions. Therefore, nowadays, the Drude–Lorentz model, discussed in the previous section, is mainly used in the case of ellipsometric studies of plasmonic materials [145,158,159,160,161,162]. For greater freedom with respect to the component describing the contribution of interband transitions, some authors use critical points, as in the case of Ag-Au alloys [82,161], or Gauss-oscillators, for Ag-Al alloys [163], instead of a Lorentz oscillator model. The comparison of the results when critical points of the M1 and M2 type model and the Drude–Lorentz model were used [82] shows that the two models have identical values. It is still unclear to what extent the changes in the *d*-band in the case of silver and *p*-block alloys can determine the dispersion pattern. In the case of Ag-Al alloys [163], Gauss-oscillators gave better results for the consideration of the interband transitions for the silver-rich alloys, while for the aluminum-rich ones the Lorentzian oscillators were more appropriate.

### 3.2. Electron Energy Loss Spectroscopy (EELS)

The Electron Energy Loss Spectroscopy (EELS) is one of the first techniques, which showed the presence of plasmons in aluminum [164]. In subsequent works, Powell investigated the EEL spectra of Be, Cd, Sb, Sn [165]. The main information in the publications is related to the bulk plasmon oscillations of these metals, located in the UV spectral region.

EELS allows the acquisition of information about the optical properties of plasmonic materials in almost the entire optical range (the graph in Figure 5b). The low-loss spectrum contains the zero-loss peak (signal from all the electrons which did not lose a measurable energy), as well as the phonon and plasmon peaks, and contains information about the band structure and the dielectric properties of the sample. It is also possible to divide the energy spectrum in momentums for a direct measurement of the band structure. By providing information about the energy loss function Im [1/ε], the method gives better visualization of the longitudinal oscillations of plasmonic waves and determination of the bulk and surface plasmon modes, which have only a longitudinal component, and are difficult for observation in the absorption spectra. In the cases of the optical characterization of nanostructures, the energy loss in the low-loss spectrum must be up to 50 eV. The spectra at higher energies are related to electron interactions in the deeper electronic levels and are not subject to the present review. A main requirement for performing the measurements is that the sample must be so thin that the electron beam can pass through it.

The EELS studies determine one of the directions for the optical characterization of materials with plasmonic frequencies in the UV spectral region. The method is particularly suitable for *p*-block metals, which have higher plasmonic frequencies than noble metals [166], a row of semiconductor materials, suitable for interband plasmonics [167], and graphene [168], whose plasmon frequency falls into the deep and extreme UV spectral ranges, where the usage of ellipsometry and UV-Vis spectroscopy is complicated.

Another EELS direction is the characterization of plasmon modes in nanostructures containing lower frequencies, falling into the visible and IR regions of the spectrum. The use of EELS as a technique for the characterization of 2D photonic and plasmonic structures [169] and determination of their surface plasmon modes was first reported in 2009. An extensive review of the study of LSPR in metal nanoparticles by EELS can be found in [170].

Typically, the localized surface plasmon covers several tens of nanometers from the metal’s surface due to the delocalization of the excitations, thus Transmission Electron Microscopy (TEM) and Scanning Transmission Electron Microscopy (STEM) platforms are used to measure the electron energy loss spectra. The combination of these microscopy techniques and EELS provides a high enough resolution for studying sub-10 nm nanostructures [171,172,173], and, respectively, the determination of the precise quality factor of the LSPR (Q_LSPR_) and its distribution. Such investigations have been reported for Cu, Ag and Au nanostructures [169,171,174]. The combination of EELS with TEM’s high resolution also allowed the tracing of LSPR by determination of the electrons’ orbital moments on the surface of silver nanodisks and nanorings [175].

The high resolution and the interaction with the electron gas in the nanostructures has been used for the direct observation of plasmonic modes in the case of nanoparticles of Ag, Au, In, Au-Ag, and charge transfer tracking (e.g., hot electrons) in plasmonic nanoparticles and heterostructures [176,177]

In Ref. [178], the complex permittivity of Au-Ag, Au-Pd and Ag-Pd alloy nanodisks on SiN_x_ is determined by applying the Kramers−Kronig method to the experimental EEL spectra. In the same article, the spectral position of the dipole, quadrupole, hexapole, and LSPR modes as a function of the Au fractional content is registered thanks to the method’s high resolution.

Nowadays, the EELS method is sensitive enough for the studying of surface plasmons of ultrathin metal films with a thickness of a few atomic layers [179]. The authors show the change in the plasmon frequency of ultrathin Ag and Au layers depending on the number of atomic layers and the crystallographic planes.

Today, the development of the technique allows combining EELS with other methods, for example, near-field imaging and Single Particle Spectroscopy, examples of which we will consider later. In Refs. [179,180], the possibility for the realization of plasmon structures mapping and visualization of the localization of the electron plasma through EELS in combination with electron tomography is shown, by taking the loss spectra of the tested sample at different rotation angles.

### 3.3. UV-Vis Spectroscopy and Dynamic Light Scattering

Here we will briefly discuss two other spectroscopic techniques, UV-Vis spectroscopy and Dynamic Light Scattering (DLS), which can provide useful information about the electronic processes in plasmonic materials.

The UV-Vis-NIR spectroscopic techniques allow the determination of the changes in the intensity of light upon interaction with nanostructures, and the acquired information can be about the reflected, transmitted and absorbed light through the sample. It is also one of the first spectroscopic techniques used for studying the optical properties of plasmonic materials. Since the first plasmonic materials were metals, the measurement of the reflection spectra was one of the first techniques for their characterization [181]. There is a huge number of papers containing data about the UV-Vis spectroscopy nowadays.

With the increasing interest in nanoparticles, works about measurements of the transmittance and absorption of solutions appeared. The UV-Vis transmittance measurements find even wider application when the plasmonic response of free nanoparticles and their concentration in solutions are studied. The method is also widely used for the determination of the boundary thickness, at which the metallic thin films begin to transmit light and for tracking the spectral position of the absorption band caused by LSPR [182,183].

A major drawback of the UV-Vis method is the sharp increase in the experimental error when determining the optical constants of thin films with thicknesses smaller than 30 nm through spectrophotometric measurements [184], which suppresses the use of this technique in studying electronic processes. More important in this direction is the possibility for the combination of this technique with Single Particle Spectroscopy (SPS), presented later in the review.

The Dynamic Light Scattering (DLS) estimates the particle size distribution by analyzing the intensity fluctuations of scattered light. The main drawback is that in order to determine the particle size distribution based on Rayleigh scattering, it is necessary to know the complex permittivity of the material from which the nanoparticles are synthesized. This somewhat limits the method to studying nanoparticles only from materials for which this information is available.

Other methods, such as fluorescence spectroscopy and Raman spectroscopy, can also independently provide information about the interaction of an analyzed substance with a plasmonic substrate by the enhancement of a fluorescent emission or Raman scattering.

### 3.4. Near-Field Imaging

The theoretical foundations of near-field imaging were laid in 1928 by E.H. Synge [14], who offered a subwavelength aperture in a metal film to be used in order to break the diffraction limit imposed by the Rayleigh criterion. With the progress in Atomic Force Microscopy (AFM) and Scanning Tunnelling Microscopy (STM), the near-field techniques were intensively developed in the mid-1980s by the integration of local probes, inspired by [185].

The most widely used near-field imaging technique is the Near-field Scanning Optical Microscopy, also called Scattering Near-field Optical Microscopy (SNOM). SNOM provides a direct image of electromagnetic fields at the nanoscale, including the plasmonic modes.

During their investigations on near-field microscopy, L. Novotny and B. Hecht [186,187,188,189,190] observed that the interaction of the metallic tip with thin films made of gold or silver excites light emission, which propagates in the direction of the surface plasmon resonance angle [17]. The individual surface inhomogeneities also influence the emitted radiation, which can be used for the acquisition of detailed information about the surface plasmon scattering, reflection, and interference phenomena. A detailed description of the basic principles of operation of modern near-field microscopy and the role of the nanoantenna effect are summarized by L. Novotny in Ref. [60].

The sensitivity of the method is determined by the type of surface under investigation and the presence of local variations in it, which can add disturbances into the oscillations of the tip [191]. The quality of the tip is of great importance, as when a tip with an aperture is used, its roughness plays an important role in obtaining an image of the surface [192].

SNOM can reach spatial resolution of ~10 nm, and in some special cases, such as at extremely low temperatures, even smaller than 1 nm [193,194,195,196]. This allows direct observation of plasmon modes in nanoparticles, thin films or nanostructured surfaces [197], as well as the determination of the chemical state and registration of different phases in nanostructures of intermetallic compounds [198]. The fast response of SNOM allows the recording of fluorescence emission with a very short lifetime of about 300 ps in aggregates of Ag nanoparticles [196].

The technique can be combined with different spectroscopic methods, such as EELS, by which information about the spatial distribution of the electromagnetic fields of plasmonic nanoparticle can be obtained [199]; and THz spectroscopy for gathering information about the dielectric function on the surface [200].

### 3.5. Single Particle Spectroscopy

The method was developed in the 2005–2010 period on the basis of EELS in TEM [201]. Its advantage is that it gives direct information for individual particles and removes the averaging from an entire ensemble of particles, seen when other techniques are used. The main disadvantage is the need for significant control of the signal–noise ratio and a careful determination of the background. The investigated particles must be well-dispersed and free from aggregation. The technique includes scattering, absorption, and extinction spectroscopy [202].

Extinction spectroscopy is mostly performed in combination with SNOM, as an excitation of the fluorescence signal by individual nanoparticles is observed during their interaction with the tip [203]. The method gives information not only about the nanoparticles’ emission, but also about the excitation of hot carriers in metallic nanoparticles and charge transfer from them in the case of catalytic reactions, SERS and Tip-Enhanced Raman Spectroscopy (TERS) [204,205]. The ability of the method for recording the fluorescence spectrum of a single molecule [206] or virus [207], located on the metal nanoparticle, is of particular importance for sensorics and biodiagnostic techniques.

Absorption spectroscopy also works in combination with SNOM. It provides information on the interaction of the electronic gas in the nanoparticle with light [208].

When a combination of SPS with EELS is used, mapping surface plasmons on a single metallic nanoparticle can be performed [209]. The possibility for the determination of the surface distribution of different multipole moments of LSPR in the spectra of silver triangular nanoprisms is demonstrated in [210]. The authors found the different modes at the three distinct positions of the nanoprisms—1.75 eV—in the triangle’s corner, 2.7 eV on the triangle’s edge and 3.20 eV in the triangle’s centre.

### 3.6. Interferometric and Polarimetric Microscopy

The principle of operation of these methods is based on the effect of changing the polarization of light upon interaction with plasmonic structures or engineered arrays of plasmonic elements, also called metasurfaces. These techniques are relatively rarely used for the characterization of plasmonic materials [210]. On the basis of the determination of Stokes parameters from polarimetric measurements, changes in amplitude, phase, direction, and polarization states of electromagnetic waves upon interaction with plasmonic nanostructures can be determined depending on their shape, size, and the material from which they are made of [211,212,213,214,215]. By offering a high degree of sensitivity for both phase and amplitude, the methods are competitive for the tracing of small refractive index changes. A disadvantage of these methods is their lower resolution compared to the near-field imaging. The main feature of these methods is the ability to control the propagation and polarization state of light.

The first studies on the potential use of metasurfaces date back to the beginning of our century, when the effect of changing the polarization in nanostructures of noble metals (silver or gold) was achieved by using the nanoantenna effect in which the light interacts with the plasmon modes [216].

At a later stage, the idea of using ultrathin plasmonic metasurfaces, acting as quarter-wave metasurface plates, to convert linearly polarized light into circularly polarized light was discussed in [217]. L-shaped antenna arrays are mostly used, although in recent years the number of reports on structures with different geometries has been increasing [218,219,220,221,222]. A full theoretical analysis of the operation of the L-shaped antenna arrays, formed by two perpendicular gold nanorods and confirmed by experimental results that show the possibility of rotating the plane of polarization and converting linearly polarized light into circular, can be found in the work of Black et al. [223].

### 3.7. Deep Learning-Based Characterization

Deep Learning (DL)-based approaches are increasingly being used nowadays. They can increase the sensitivity of optical recording hardware, as well as significantly improve the effective sensitivity by extracting nuanced spectral or graphical features from noisy data [224,225].

DL helps to overcome the difficulties of the conventional methods, e.g., by quickly solving the Maxwell equations during the analysis of a given structure in order to determine its spectral response, and more importantly, by solving the even harder inverse problem, when plasmonic structures have to be modelled from a preliminary given desired spectrum. These complex calculations are performed by self-learning computational models called artificial neural networks (ANNs) [226].

ANNs are built of interconnected software units (nodes), organized into multiple layers. The transfer of the signals from one node to another and the layers’ arrangement are of great importance. When ANNs are used for the modelling of nanostructures, the used models are the Geometry-Predicting-Network (GPN), which predicts the nanostructure’s geometry on the basis of a given spectrum and the Spectrum-Predicting-Network (SPN), which gives the spectrum for a given geometry of the nanostructure [226,227,228].

By training neural networks on large data sets, subtle variations in the modelling of plasmonic nanostructures, which would otherwise be lost in traditional analysis, can be identified and interpreted [229,230]. The success of these methods for the prediction and production of reliable inferences about small changes in plasmonic responses is highly dependent on the quality and diversity of the training data, along with the architecture of the neural network [231,232].

The main challenge for the development of DL systems is the downsizing of the AI-supported SERS diagnostic systems and the ability to create mobile devices.

## 4. Plasmonic Materials and Approaches for Design of Their Electronic States

The electronic structure of plasmonic materials is decisive for the excitation of hot electrons and therefore an important factor in determining the application of plasmonic materials in the fields of photocatalysis, sensorics and surface enhancement spectroscopies. In this section, we will consider the methods for the tuning of the plasmon frequency of various materials by changing their electronic states, including change in interband transitions and charge carrier injection.

The growing interest towards intermetallic alloys and the synthesis of various new plasmonic materials, such as graphene and MXenes, opens up possibilities for the tuning of plasmons to a desired frequency.

### 4.1. Noble Metals and Their Alloys

The role of charge transfer in plasmonic materials has been increasingly studied in recent years with a view to its practical application in various areas of photocatalysis, such as carbon reduction [233] or hydrogen production [234,235], as well as in the field of SERS [236,237,238], in order to increase the sensitivity and selectivity of existing methodologies or to create new ones for medical or molecular biodiagnostics.

Usually, the heterostructures of noble metals with oxides are used to absorb light from the UV or IR part of the electromagnetic spectrum and to generate additional carriers for enhancement of the electromagnetic field at hot spots in nanostructures for photocatalysis and SERS, as some of the most famous examples of heterostructures being Ag-SiO_2_, Ag-TiO_2_ and Au-TiO_2_ [229,230,231,232,233,234,235,236,237,238,239,240,241,242,243,244].

The photosensitivity of a material, TiO_2_ for example, when irradiated with UV light, causes the excitation of electrons in its conduction band, and as a result, a significant charge transfer occurs, and a chemical interaction takes place between the SERS substrate and the analyzed molecule [245]. A chemical enhancement in the SERS performance of a Ag/TiO_2_/R6G (Rhodamine6G) system is reported in [245], at which an electronic transfer of hot Ag electrons is realized from the Fermi level to the LUMO of R6G, caused by a combination of the energies of the valence and conduction bands of TiO_2_ and the LUMO of R6G. In [246], a metal/ferroelectric hybrid system of Ag/BiFeO_3_/carbon nanofibers was used, and the IR irradiation was transferred into pyroelectric charge, which adjusts the electron densities of Ag and thus enhances the electronic fields in the “hot spots”.

Since silver and gold are the most effective materials for SPR and LSPR excitation in the visible spectral region, the Ag-Au alloys were one of the firsts investigated towards plasmonic properties. Studied in the 1990s, the Ag/Au and Au/Ag core–shell structures were one of the first investigated intermetallic configurations [247,248,249], due to the combination of gold’s chemical stability and silver’s high efficiency for surface plasmon excitation in them [250]. A similar effect was also observed in other alloys, such as Au-Cu, Au-Pd and Ag-Pt; therefore, at the same time, a number of reports regarding the formation of intermetallic clusters and the study of their absorption spectra appeared [251].

The electronic structure of the intermetallic clusters of noble metals, together with their shape and the atomic distribution of the different metals in them, determine their absorption energy and chemical interaction with the environment [99], and therefore they are subjected to a wide investigation both in practical experiments [252] and theoretical modelling [253].

**Figure 6:**
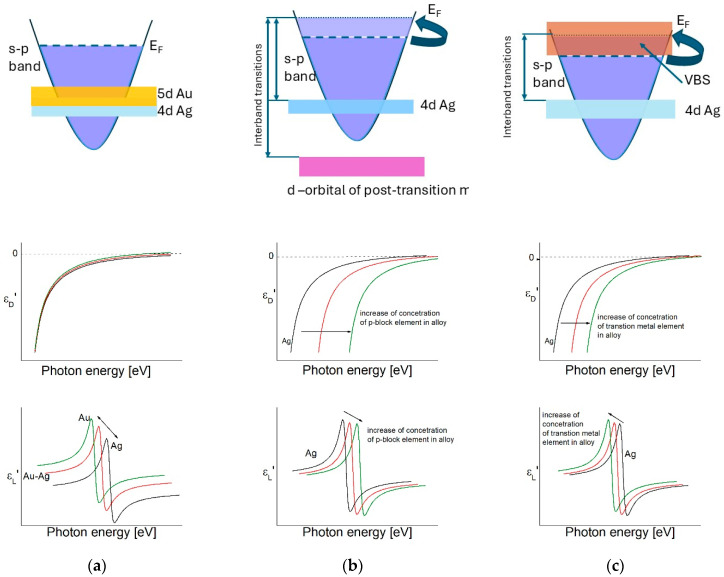
Schematic representation of the band structure and the expected behaviour of the Drude and Lorentz components (presented as one oscillator) of noble metal alloys: (**a**) alloys of two noble metals (Ag-Au). The relatively equal free electron concentration does not change the position of the Fermi level and has a weak influence on the Drude component ε_D_′, while the overlapping of the *d*-bands of the two metals leads to variation in the dispersion of the Lorentz component, ε_L_′. (**b**) Alloys of silver with *p*-block metals. The larger number of free electrons in the p-block metals increases the Fermi energy and the plasmon frequency of ε_D_′, while the increased energy for interband transitions from the 4*d* band of silver causes a shift in the ε_L_′ maximum towards the higher photon energies. (**c**) Alloys of silver with transition metals (Fe, Co, Ni). The increase in the free electrons causes changes in the Drude component similar to those for the *p*-block intermetallic alloys, while the vacant *d*-band of the transition metals forms virtual band states (VBS) around the Fermi level and therefore the 4*d* Ag band gets closer to it and the ε_L_′ maximum shifts towards the lower photon energies. The band structure and complex permittivity are adapted from data given in [71,81,82,105,151].

The main principle of tuning in the case of intermetallic Cu, Ag and Au alloys is that the energy for transitions from the *d*-band can vary between these of the two noble metals (Figure 6).

**Figure 7:**
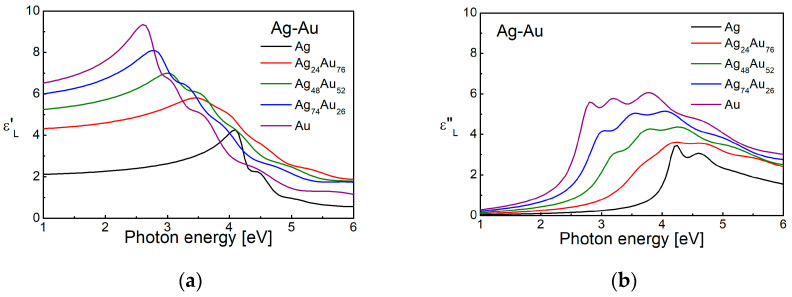
Real (**a**) and imaginary (**b**) parts of the Lorentz component in the Drude–Lorentz model, describing the interband transitions from the *d*-bands of Ag and Au and their shifting in the Ag-Au alloys due to the overlapping of the *d*-bands of the two metals. Data, published in [82], were used for the calculations.

The Lorentz component of the Drude–Lorentz model, determined using the parameters published in [82], is shown in Figure 7. It can be seen that the ε_L_ values for the Ag-Au alloys take intermediate places between those of silver and gold. The similar crystal lattices of gold and silver allow the preparation of Ag-Au alloys in a continuous row of solid solutions throughout the whole interval of concentrations between the two metals. This determines one of the advantages of using Ag-Au nanostructures—the ability for smooth variation in the properties, including the plasmon frequency. It has been shown in [161], that on the basis of the obtained dependences of the parameters of the Drude–Lorentz model, determined by ellipsometric measurements, one can predict the optical properties and choose the exact composition of a Au-Ag alloy with a maximum efficiency for the excitation of LSPR for a specific wavelength, which falls in the spectral interval between the plasmon frequencies of silver and gold. The possibility for variation in the transition energy of the *d*-band in the Cu-Ag heterostructure and thus controlling the energy of thermal electrons at the Fermi level was recently discussed in [254].

It can be seen that the plasmon frequency coincides with that of the interband transitions in all alloys between noble metals (Cu, Ag and Au), i.e., hot electrons and hot holes are excited at the same frequency, which in some cases can be a serious drawback. This problem can be solved by alloying the noble metals with some post-transition metals.

Silver and gold alloys with *p*-block metals have been the subject of research for plasmon frequency tuning since the early 1970s and the early 1980s [162,255]. Solid solutions based on silver and gold were extensively studied during this period with the aim to shift the plasmon frequency of these noble metals towards higher photon energies (Figure 6b).

**Figure 8:**
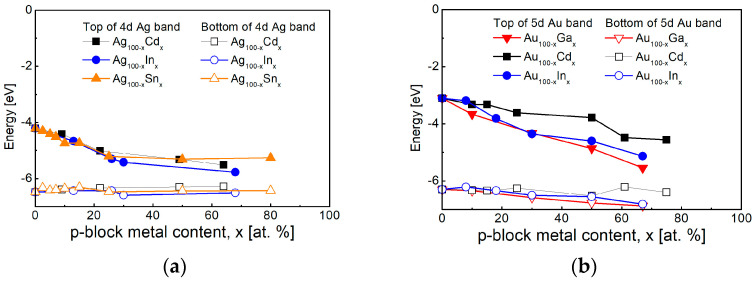
Top and bottom of 4*d* Ag and 5*d* Au bands, according to published data from XPS analysis for (**a**) Ag_100−x_Cd_x_ [256], Ag_100−x_In_x_ [256] and Ag_100−x_Sn_x_ [257]; and (**b**) Au_100−x_Ga_x_ [258], Au_100−x_Cd_x_ [258] and Au_100−x_In_x_ [258] systems. Fermi energy is set to be zero (E_F_ = 0 eV).

The research of the intermetallic compounds is focused on their electronic structure because of their good conductivity. A significant number of studies using X-ray Photo-electron Spectroscopy (XPS) show an increase in the energy gap separating the *d*-band and the Fermi level, as a result of a shift in the *d*-band of silver and gold [256,257,258]. Data from various studies are presented and summarized in Figure 8. It can be seen that the peak of the 5 Au *d*-band is significantly more strongly influenced by the *p*-block metal content compared to that of silver, and in both cases the position of the bottom changes less.

A major problem for plasmonics in the UV spectral region is that the significant optical losses related to interband transitions in noble metals make them ineffective in this part of the electromagnetic spectrum [68]. This is the reason for the increased interest in recent years of searching for materials that have a better efficiency of surface plasmon resonance excitation in the UV spectral region. A number of studies have been focused on post-transition (*p*-block) metals and their alloys with Ag and Au, because of their higher plasmon frequency caused by the larger number of valence electrons in them [68,72], as well as on some transition metals, such as rhodium, palladium, platinum and chromium [70]. The higher efficiency for the excitement of the surface plasmon resonance of *p*-block metals can be improved by their alloying with noble metals [71]. Special attention has been paid to the intermetallic compounds from such binary systems in recent years [73,74].

The plasmon frequency and interband transitions can be separated in intermetallic compounds of silver and gold with *p*-block metals and a separation of the excitation energies of the hot electrons and holes can be realized this way. Studies of polycrystalline films containing intermetallic compounds of gold with In, Sn and Sb show a shift in the plasmon frequency in the UV region in the range of 5–8 eV [74,259,260,261,262], and possibility for LSPR excitation in the interval 3–5 eV. At the same time, a second point in the visible region is observed in the case of thin films containing intermetallic phases of AuIn_2_ [259,260], AuSn [261] and AuSb_2_ [262], due to interband transitions of 5*p* electrons of the post-transition metals. As a result of the shift in the 4*d* Ag band in the intermetallic compounds of silver with Cd and In—AgCd, Ag_3_In and AgIn_2_, a decrease in the imaginary part of the complex permittivity is observed in the spectral range 3–6 eV [74,159,263]. The plasmon frequency of these compounds also falls in the interval 7–12 eV, and, at the same time, transitions from 5*s* Cd and 5*p* In electronic states are observed in the spectral range 1–3.5 eV.

Some optical characteristics of noble metals, post-transition metals and their intermetallic compounds are compared in Table 2. It can be seen that the post-transition metals and their intermetallic compounds possess higher ω_s_ values, which fall into the UV. Respectively, the Q_LSPR_^MAX^ values even though relatively low, also fall in the UV spectral region in the case of intermetallic compounds. The noble metals and the intermetallic compounds show Q_SPR_ values higher than 10, which increase in the IR spectral region, but an increase in ε″ is observed at the same time, which leads to a decrease in the propagation length of the plasmonic waves, as, in some cases, the decrease in the imaginary part in the UV spectral region leads to the appearance of a second region with higher Q_SPR_ values.

Unlike p-block metals, rare-earth and transition metals, in which the last *f* and *d* orbitals are not completely filled, have intense interband transitions in the visible and infrared spectral regions, which reduces their efficiency [71]. The increase in the free charge carriers causes shifting of the LSPR peak of gold towards the higher photon energies in the visible spectral region. The alloying of Fe, Co and Ni with silver and gold is studied in Refs. [105,264,265]. The nanoparticles of these alloys are interesting because of their magnetic properties and their application in biodiagnostics [226]. The main problem is that the effective magnetic properties are acquired at higher concentrations of transition metals, but the effectivity of the excited LSPR is lost at the same time.

### 4.2. Two-Dimensional Plasmonics

#### 4.2.1. Graphene and Graphene Oxide

Novoselov’s results, published in 2004, on the synthesis of graphene opened up a whole new direction in the field of creating new materials for plasmonics [266]. The new material was found to be a two-dimensional semimetal with a tiny overlap between the valence and conductivity bands. The subsequent EELS studies have shown that the bulk plasmon (~5 eV) of graphene is associated with the bulk plasmon of graphite and originates from the collective excitations of the π valence electrons (π plasmon at 7–12 eV) and all valence electrons (more intense and broader σ + π plasmon at 28–33 eV). The EELS showed that the π mode at 5.1 eV in single epitaxially grown graphene (EG) shifts to 5.6 eV for the bilayer and to 6.3 eV for 3–4 layers EG [267].

The intraband plasmon of graphene is of greater practical interest. The long propagation length of the plasmon’s wave is the reason why graphene is studied as a potentially important material for plasmonics. The lower concentrations of electrons and holes *N*_c_ ~1.0 × 10^12^ cm^−3^ suggest that its plasmonic activity is in the IR and THz spectral regions [268]. From the perspective of the practical application of graphene’s plasmonic properties, the scientific interest is directed towards the shifting of its plasmon frequency to the shorter wavelengths, falling in the near-infrared and visible spectral regions. Various approaches for charge injection are used, such as the applying of extremely high voltage [168], doping or formation of heterostructures with various metals or Ag/Au nanoparticles [269].

Tuning of the graphene’s plasmon frequency was achieved for the first time in 2021 [270,271] by the quantum confinement of charge carriers and reduction in losses. The authors checked their results through SERS of CuPc (copper phtalocyanine) at a wavelength of the laser irradiation of 633 nm [270].

From the point of view of effectivity in the visible region, graphene oxide finds many applications in SERS in combination with the nanostructures of noble metals (Ag, Au) [236,272,273,274]. An advantage of using graphene oxide is that its Fermi level can be flexibly manipulated by adjusting the direction of the ferroelectric polarization or the temperature of the ferroelectric substrate, and, respectively, tuning the resonance enhancement of the Raman signal [275,276].

#### 4.2.2. Plasmons in Semiconductors—Transition Metal Chalcogenides

An extensive review of the plasmonic properties of natural materials—metals, semiconductors, oxides, halides and others, has been recently presented in [68].

Here we will consider only the properties of a group of semiconductor materials, characterized by plasmonic properties in the IR region and of interest in the field of 2D materials, which have a higher concentration of free charge carriers [277].

In this group of materials, as in graphene, plasmonic properties related to interband transitions can be expected, with a plasmonic frequency falling in the UV spectral region, as shown by DFT calculations [278,279], EELS data [280,281,282,283] and Spectroscopic Ellipsometry [284].

Special attention should be given to the group of naturally hyperbolic transition metal dichalcogenides. The first theoretical calculations using DFT [278,279] show that the 1T phase of Ta, Nb, Ti, Co, Hf, Ru and Zr dichalcogenides possesses hyperbolic properties in the IR spectral region, i.e., as triaxial anisotropic crystals, they possess dielectric properties (their complex permittivity has positive values) along two of their optical axes, while along the third axis they have a metallic character (negative value of the complex permittivity).

The ellipsometric analysis of anisotropic media is a task of great difficulty. However, the results in Ref. [67] show a change in the sign of the in-plane complex permittivity in the case of Ta-based compounds—TaS_2_ and TaSe_2_ at wavelengths of ∼1110 nm (∼1.11 eV) and ~1217 nm (~1.01 eV), respectively. In the case of NbSe_2_, the authors observe that the crystal loses its anisotropic properties at a wavelength of 1390 nm, where ε changes its sign and has negative values at the longer wavelengths.

In the case of materials with positive values of the real part of the diagonal dielectric tensor, such as hybrid structures of MoS_2_, MoSe_2_ and WSe_2_ with gold [67], various techniques are used, which aim to increase the concentration of free carriers so as to exhibit plasmonic properties in the visible and near-IR regions [285,286,287].

#### 4.2.3. MXene

The combination of the interest towards TiN, as a plasmonic material with properties similar to those of gold in the IR spectral region, and towards graphene led to the idea of obtaining carbides and nitrides in a layered form. The first MXene was reported in 2011 [288,289]. The MXenes are successively alternating layers of carbon (or nitrogen) atoms and transition metals (Sc, Ti, V, Cr, Y, Zr, Nb, Mo, Hf, Ta, W) or rare earths (Lu) [290] designated by the general formula M_n+1_X_n_T_x_ (where M and X denote the metal and carbon (nitrogen) element with a number of layers, n; T_x_ is used to describe the diverse surface terminations) [64]. The MXenes undergo termination by chalcogenide or halogen elements, as the most preferred are oxygen and fluorine, as well as OH groups. Among all MXenes, the Ti_3_C_2_ receives the most attention because of the presence of developed synthesis and processing methods, its high conductivity and pronounced plasmonic response. An interesting sub-group of MXenes are the MAX phases, written by the general formula M_n+1_AX_n_, in which the A denotes a post-transition element (Al, Si, P, S, Ga, Ge, Cd, In, Sn, Tl, Pb). There are reports in the literature of about 30 MXene structures composed of two transition metals, such as (Ti_0.5_Nb_0.5_)_2_C, (V_0.5_Cr_0.5_)_3_C_2_ [289].

The study of MXenes’ optical properties by absorption spectroscopy and Spectroscopic Ellipsometry [141,142] show that the real part of their complex permittivity possesses positive value in the visible spectral region, and then changes its sign in the infrared, as the values of the imaginary part in the IR spectral region are lower than these of noble metals.

There are several techniques for tuning the plasmon frequency in the IR spectral region, as well as the frequency and modulations of electronic band structure in MXene structures: applying a voltage in different directions [291,292], charge transfer from quantum dots [293] or heterostructures with noble metals [294], and doping with *p*-block elements [295].

By changing MXene film’s composition, the epsilon-near-zero (ENZ) point, where the optical properties change from dielectric to metallic, varies in the spectral range from 1.1 to 2.6 µm [142]. The results for some of the most investigated MXenes, Ti_3_C_2_ and Ti_3_C_2_T_x_, show that the optical response of the MXene film is dominated by a pronounced Drude behaviour from free charge carriers in the infrared spectral range. The epsilon zero points of the real part of complex permittivity of Ti_3_C_2_T_x_ and Ti_3_C_2_ are located at 800 nm and 1418 nm, respectively. Several absorption features related to interband electronic transitions at higher photon energies determine the absorption of the MXenes structures in the NIR−Vis−UV region [296]. Depending on the transition metal, the band gap can vary from 0.33 eV up to 1.55 eV for Ti_2_AlN and Ti_3_C_2_, respectively.

Density Functional Theory (DFT) calculations show that the *d*-band of the transition metal participating in the MXene layer structure has an important role in determining the interband transitions in the NIR−Vis−UV region and the plasmon frequency [291].

The DFT calculations of the electronic structure of Ti_2_C and Ti_3_C_2_ monolayers, presented in [297], show that the lowest valence bands from 12 eV to 10 eV are formed by the C-*s* states with a small mixture of Ti-*p* and *d* states. A strong hybridization of Ti-*d* and C-*p* states (Ti–C bond) form higher valance bands from 5.5 eV to 2.5 eV. The authors found that the surface terminations using the F, O, or OH group result in a shift in this band into the lower energy range.

Zhang, Y. et al. [295] studied theoretically the modulations of the electronic band structure and the band gap of Hf_2_CO_2_ MXene by the first principles of Density Functional Theory. They established, by substitution-doping approaches (two different substitution sites, i.e., C and O sites) and various *p*-block dopants, that the Si_C_-, Ge_C_-, BN_C_-, and NF_O_-doped Hf_2_CO_2_ nanosheets possess wide band gap semiconductor properties, while the N_C/O_-, B_O_-, P_O_-, and F_O_-doped Hf_2_CO_2_ nanosheets go to semiconductor or metallic states.

The plasmonic properties of the materials discussed in Section 4.2 are summarized in Table 3. Their plasmon frequency, ω_s_, except for TiN and HfN, is situated in the IR spectral region. All of them are characterized by low Q_LSPR_ values, with a maximum value not exceeding 1, except for nitrides.

### 4.3. Future Challenges–2D Noble Metals and Their Intermetallic Compounds

By following the trends for obtaining new 2D materials and their unique properties, the question of the synthesis of noble metals and intermetallic alloys in such a form arises. Accordingly, the research contributes to the preparation of new materials with new properties, as well as to the miniaturization of plasmonic devices. This new direction has been gaining ground since 2015, with the first reports being about the obtaining of nanosheets from Pd [300], Pt [301] and Rh [302]. The extensive review of Chen, Y. [300] about the present state of 2D noble metals and intermetallic compounds shows that the scientists present mainly synthesis under the form of nanosheets.

The preparation of 2D platinum intermetallic alloys is presented in [303]. These materials are interesting due to their application in the field of photocatalysis. The possibility for the synthesis of 2D intermetallic alloys of platinum is determined by the fact that most of the obtained compounds—PdCd, PdZn, PdSn, etc., possess a hexagonal crystalline structure, which facilitates their formation in nanosheets.

However, this field is still in its infancy. In the last 2 years, a number of reports on the preparation of 2D metals with thickness falling in the ångström range or in a single atom layer form appeared.

The main problem for obtaining 2D noble metals is their crystalline structure and surface energy [304]. The high surface energy supports the growth of noble metals in 3D structures when they are deposited on a dielectric substrate and is the reason for the poor adhesion of the metal coating. When very thin layers of silver and gold are prepared, they disassociate by forming island structures or nanoparticles. This effect is particularly strong when trying to deposit very thin films of noble metals with thicknesses of a few atomic layers by conventional deposition techniques and is the reason for the research of approaches for the stabilization of 2D noble metals.

The 2D dichalcogenide materials and MXene structures, discussed in the previous sections, help in this synthesis. Preparation of 2D metals (including Bi, Ga, In, Sn and Pb) at the ångström thickness limit by encapsulation between two MoS_2_ monolayers was reported in Ref. [305].

Of course, the challenge of obtaining the most famous and widely used plasmonic materials—gold and silver—lies in this direction. Since both metals tend to grow in 3D, this can be a certain obstacle to go down to the monolayer form. However, the techniques used for the synthesis of these metals in the form of nanoparticles allow them to be obtained in the form of nanodisks, nanosheets, and nanoplates with a smaller thickness [301]. As an example, the synthesis of 2D silver nanosheets with a nominal thickness of 4.4 ± 0.3 nm was presented in [306].

In the past year, 2024, the first results for the successful production of few-atom-layer silverene (called like that in association with graphene) [307] and a single-atom-layer gold [308] were published. The authors of Ref. [308] used a Ti_3_C_2_ MXene for the basis, in which they grew a single Si layer to form Ti_3_SiC_2_ MAX and then substituted the silicon with gold to obtain a Ti_3_AuC_2_ phase, which by itself represents a monolayer of gold (called goldene) encapsulated in the Ti_3_C_2_ layered structure.

Recently published theoretical DFT calculations [309] show that the goldene remains in a stacking configuration for up to six layers, after which it spontaneously transitions into a bulk-like gold structure, adopting a rhombohedral (ABC-like) stacking characteristic of bulk face-centred cubic (FCC) gold. This suggests serious change in the electronic structure when the limiting number of golden layers is reached. The authors also point out that the monolayer of goldene exhibits anisotropic optical absorption, which is absent in the bulk gold.

These estimations were confirmed experimentally in [308]. The as-prepared Au monolayers stacked in the Ti_3_C_2_ MXene coalesce when the goldene–goldene interlayer distance got below 7 Å. At a larger distance of 10 Å this undesired effect significantly fades. The presence of inclusions of other materials can also cause coalescence of the Au layers. The usage of a MAX phase increases the goldene–goldene interlayer distance and slows down the coalescence process.

## 5. Conclusions

The development of plasmonics represents a series of theoretical and experimental achievements. The present review shows that materials, whose spectral range and efficiency are determined mainly by their natural properties, were used in the initial stage. With the development of plasmonics not only for the visible, but also for the IR and UV parts of the spectrum, the need for finding new materials with the necessary efficiency of surface plasmon excitation in a desired spectral range increases.

The 2000–2010 period is a milestone, as a number of new materials, such as conductive oxides, graphene and transition metal dichacogenides, began to enter the picture and find application in plasmonics. The research in the field of electronic processes is of great importance.

Various experimental techniques are available for this purpose. Since the complex permittivity of the materials is a function of the electronic states in them, methods such as Spectroscopic Ellipsometry and EELS, which allow its determination, are widely used in for studying and modelling of plasmonic materials. The development of new methods, such as SNOM and Single Particle Spectroscopy, makes it possible to directly observe the behaviour of electrons in plasmonic nanostructures and nanoparticles. Although still in their early stages, the methods for Deep Learning-based characterization are increasingly entering into practice.

The electronic structure of the material is a fundamental factor determining the hot electrons’ kinetic energy and the possibility for their transition to bonding or antibonding orbitals, and thus determining the chemical interaction between the nanostructures and the analyte. The possibility for the control of the electronic structure allows achievement of good selectivity of the metallic nanostructures and determines their interaction with external substances. This is of particular importance for various surface enhancement spectroscopies, which are increasingly used for early medical and molecular diagnostics.

Depending on the material from which the plasmonic nanostructures are made, different approaches are used for the control of the electronic processes in them. The main approach for tuning plasmonic nanostructures is by the manipulation of the interband transitions from their *d* band.

In the case of oxides, nitrides and graphene, the most appropriate approach is the charge transfer, by which a change in the free electron’s concentration is achieved. The most commonly used methods for this purpose are application of an external voltage and preparation of heterostructures with noble metals.

The synthesis of 2D graphene sets a whole new direction in the development of plasmonic materials and of course raises the question how to obtain in such form the most important plasmonic materials—silver and gold, used since the beginning of plasmonics, as well as their intermetallic alloys.

## Figures and Tables

**Table 1:** Applicability and general data of the most used methods for the evaluation of the properties of plasmonic materials in the form of thin films (TF), metasurfaces (MS) and nanoparticles (NPs): Spectroscopic Ellipsometry (SE), UV-Vis-NIR spectrophotometry, Dynamic Light Scattering (DLS), Electron Energy Loss spectrometry (EELS), Scattering Near-field Optical Microscopy (SNOM), Single Particle Spectroscopy (SPS), Interferometric Microscopy (IM), Polarimetric Microscopy (PM), and Deep Learning (DL). For more abbreviations see the List of Abbreviations at the end of the review.

Method	Measured Parameters	Calculated Parameters	Sample Type			Spot Limit	Combination with Other Methods	Additional Data
			TF	MS	SP			
SE	Ellipsometric angles (Ψ, Δ)	Optical constants Anisotropy	+ *	+ *	−	≥10 μm	UV-Vis-NIR	Thickness Homogeneity Porosity
UV-Vis-NIR	Reflection Absorption Transmittance *	Optical constants	+ *	+ *	−	>1 mm	SE SPS	Thickness Homogeneity Porosity
DLS	Scattered light intensity	Particle size distribution	+	−	+	≥1 mm		
EELS	Number of electrons passed through the sample after inelastic scattering.	Optical losses Band structure Dielectric properties SPR and LSPR modes	+	+	+ **	sub-10 nm	TEM SEM SAED	Surface image Composition Crystalline structure
SNOM	Surface image Plasmon modes Fluorescence emission		+	+	+	sub-10 nm	AFM TEM STM EELS THz spectroscopy	Surface image Chemical state Registration of different phases
SPS	SP absorption and emission. Geometry and surface of SP. Optical heterogeneity	Chemical composition	−	−	+	sub-10 nm	SNOM TEM SEM TERS	NP emission Hot carriers’ excitation in metallic NPs Charge transfer in catalytic reactions
IM	Amplitude and phase of electromagnetic waves	Polarization states Small changes in refractive index	+	+	+	λ/4		
PM	Amplitude, phase and polarization states of electromagnetic waves	Stokes parameters Anisotropy	+	+	+	λ/2		
DL	Data analysis	Materials design Signal prediction Data processing	+ ***	+ ***	+ ***		All methods	

* Depends on the absorption coefficient of the material in the measured range; ** in combination with SNOM; *** analysis of information from other techniques.

**Table 2:** Summary of literature data on the plasmonic properties (crossover point of the real part of complex permittivity (ε′ = 0), ω_s_, Drude plasmon frequency, ω_p_, maximal values of efficiency coefficients for excitation of LSPR *Q*_LSPR_^MAX^ = −ε′/ε″ and SPR − *Q*_SPR_ = −ε′/ε″ (the spectral range, in which the imaginary part of ε increases, is specified) of some noble metals, post-transition metals and their intermetallic compounds.

Material	ω_s_ [eV]	ω_p_ [eV]	Q_LSPR_^MAX^/ω(*Q_LSPR_^MAX^*) [eV]	Q_SPR_/ω(*Q_SPR_*) [eV]	ε″	Ref.
Ag	3.9	9.02	~20 * (2.0 eV)	32–2000 * (3.5–0.5 eV)	0.87–8 * (3.5–0.5 eV)	[82]
Au	2.5	8.76	~12 * (1.5 eV)	10–1500 * (2.2–0.5 eV)	2.50 (1.5 eV) 2.3–50 (2–0.5 eV)	[82]
Al		14.3	9.3 (8.6 eV)	n.a.		[69]
Ga		14.05	13.6 * (11 eV)	n.a.		[69]
In	10.9	11.7	4.60 (5.10 eV)	5.6 (3.5 eV) *	5.2 (3.5 eV) 1.7 (5.10 eV)	[151]
Ag_48_Au_52_	3.7	8.89	~6.7 (1.7 eV)	10–700 * (2.6–0.5 eV)	1.84–90 (2.6–0.5 eV)	[82]
AuAl_2_	6.7	13.4	3 (1.3 eV) *	10–80 (1.7–1 eV)*	10–80 (1.7–1 eV)	[259]
AuGa_2_	7.8	13.1	1.8 (3.36 eV) *	22–10 (3–4 eV) * 10–70 * (1.5–1 eV)	~4.5–11 (3–4 eV) ~23–38 (1.5–1 eV)	[259]
Ag_3_In	8.3	11.9	14.4 (2.2 eV) *	10-2000 * (4–0.5 eV)	1.7–6.7 (3.6–0.5 eV)	[151]
AgIn_2_	6.0	11.2	1.1 (5.5 eV) *	10–50 * (0.8–0.5 eV)	1.87 (5.5 eV) 18.0–41.3 (0.8–0.5 eV)	[151]
AuIn_2_	7.2	11.8	2 (3.4 eV) *	10–25 * (4–2.9 eV) * 10–45 * (1.4–1 eV)	3–12 (4–2.9 eV) 22–40 (1.4–1 eV)	[259]

* calculated values from data, presented in the specified references.

**Table 3:** Summary of the literature data on the plasmonic properties (crossover point of the real part of complex permittivity (ε′ = 0), ω_s_, Drude plasmon frequency, ω_p_, maximal values of efficiency coefficients for the excitation of LSPR *Q*_LSPR_^MAX^ = −ε′/ε″ and SPR − *Q*_SPR_ = −ε′/ε″ (the spectral range, in which the imaginary part of ε increases, is specified) of some transition metal chalcogenides, nitrides, MXenes and MAX phases.

Material	ω_s_ [eV]	Q_LSPR_^MAX^/ω(*Q_LSPR_^MAX^*) [eV]	Q_SPR_^MAX^/ω(*Q_SPR_^MAX^*) [eV]	ε″	Ref.
TaS_2_	1.11	~1.5 * (1.37)	~17 * (1.37)	2.97 (1.37) 7.2 (1.37)	[67]
TaSe_2_	1.01	~1.5 * (1.37)	~14 * (1.37)	3.5 (1.37)	[67]
NbSe_2_	0.89	0.8 * (0.89 eV)	~8 * (0.89 eV)		[67]
TiN	2.66	~4 * (1.1 eV)	~10–250 * (2–0.5 eV)	3.2–42 (2–0.5 eV)	[298]
HfN	3.1	~3 * (1.9 eV)	~10–98 * (2.4–1 eV)	2.5–25 (2.4–1 eV)	
Ti_3_C_2_T_x_	1.05	~1 * (0.5 eV)	~10–25 * (0.7–0.5 eV)		[142]
Ti_2_AlC	0.5 ~2.65	~1.5 * (0.3 eV)	~10–250 * (0.3–0.2 eV)	90–100 (0.3–0.1 eV)	[298]
(Ti_0.5_Nb_0.5_)2AlC	0.55	~ 0.5 * (0.2 eV)	~ 10–40 * (0.1–0.2 eV)	90–140	[299]

* calculated values from data, presented in the specified references.

## Data Availability

No new data were created or analyzed in this study. Data sharing is not applicable to this article.

## List of Abbreviations

	**General**
2D	Two-Dimensional
3D	Three-Dimensional
CRISPR	Clustered Regularly Interspaced Short Palindromic Repeats
ENZ	Epsilon-Near-Zero
FCC lattice	Face Centred Cubic lattice
HOMO level	energy level of the Highest Occupied Molecular Orbital
IR	Infrared spectrum (NIR—near IR)
LSPR	Localized Surface Plasmon Resonance
LUMO level	energy level of the Lowest Unoccupied Molecular Orbital
MS	MetaSurface
NP	NanoParticle
Q	Quality factor
SP	Single Particle
SPR	Surface Plasmon Resonance
TF	Thin Film
UV	Ultraviolet spectrum
Vis	Visible spectrum
Δ	Ellipsometric angle; difference between the phases of the reflection amplitude coefficient for p- and s-polarizations
Ψ	Ellipsometric angle; ratio of the reflection amplitude coefficient for p- and s-polarizations
	**Methods**
AFM	Atomic Force Microscopy
ANN	Artificial Neural Network
DETECTR	DNA Endonuclease-Targeted CRISPR Trans Reporter
DFT	Density Functional Theory
DL	Deep Learning
DLS	Dynamic Light Scattering
EELS	Electron Energy Loss Spectroscopy
EMA	Effective Media Approximation theory
FDTD	Finite-Difference Time-Domain method
GPN	Geometry-Predicting-Network
IM	Interferometric Microscopy
PM	Polarimetric Microscopy
SE	Spectroscopic Ellipsometry
SEF	Surface-Enhanced Fluorescence
SEHRS	Surface-Enhanced Hyper Raman Scattering
SEIRA	Surface-Enhanced InfraRed Absorption
SEROA	Surface-Enhanced Raman Optical Activity
SERS	Surface-Enhanced Raman Spectroscopy
SHERLOCK	Specific High-Sensitivity Enzymatic Reporter UnLOCKing
SNOM	Scattering Near-field Optical Microscopy (NSOM—Near-field Scanning Optical Microscopy)
SPN	Spectrum-Predicting-Network
SPS	Single Particle Spectroscopy
SPRM	Surface Plasmon Resonance Microscopy
STEM	Scanning Transmission Electron Microscopy
STM	Scanning Tunnelling Microscopy
TEM	Transmission Electron Microscopy
TERS	Tip-Enhanced Raman Spectroscopy
UV-Vis-NIR	UV-Vis-NIR spectrophotometry
XPS	X-ray Photo-electron Spectroscopy
	**Materials**
AZO	Al:ZnO
CuPc	Copper PhtaloCyanine
EG	Epitaxially grown Graphene
Goldene	2D gold (single-atom-layer)
GZO	Ga:ZnO
ITO	Indium Tin Oxide
MXene	metal (M) carbide/nitride (X) in the form of alternating layers
MAX phase	MXene with an intermediate layer of a post-transition element
R6G	Rhodamine6G
Silverene	2D silver (few-atom-layer)
